# Retrospective multicenter analysis of causes of death in wild mammals in Southern Germany (2019–2023)

**DOI:** 10.3389/fvets.2026.1805419

**Published:** 2026-04-21

**Authors:** M. Nowak, V. Herschmann, A. Blutke, B. Blazey, B. Boehm, U. Fischer, A. M. Gager, A. Gangl, C. Gangl, E. Großmann, P. Hernando, W. Huber, E. Kappe, M. Mueller, D. Nobach, J. Reichert, K. Rigbers, B. Schade, I. Schwabe, B. Strobel, M. Suchowski, M. Suntz, T. Thiele, J. Tyczka, K. Yen, K. Zetzmann, K. Feld, N. Kolb

**Affiliations:** 1 Institute of Veterinary Pathology at the Center for Clinical Veterinary Medicine, LMU Munich, Munich, Germany; 2 Chemical and Veterinary Analysis Agency (CVUA) Stuttgart, Fellbach, Germany; 3 Bavarian Animal Health Service, Poing, Germany; 4 Chemical and Veterinary Analysis Agency (CVUA) Freiburg - Institute of Animal Health, Freiburg, Germany; 5 Bavarian Health and Food Safety Authority, Oberschleißheim, Germany; 6 Bavarian Hunting Association, Feldkirchen, Germany; 7 Aulendorf State Veterinary Diagnostic Centre (STUA), Aulendorf, Germany; 8 Chemical and Veterinary Analysis Agency (CVUA) Karlsruhe, Karlsruhe, Germany; 9 Bavarian Health and Food Safety Authority (LGL), Department of Animal Health, Erlangen, Germany; 10 Institute of Forensic and Legal Medicine, University Hospital Heidelberg, Heidelberg, Germany

**Keywords:** anthropogenic mortality, Central Europe, infectious disease, necropsy, One Health, surveillance, vehicle collision, wildlife pathology

## Abstract

**Introduction:**

Wild mammals are important indicators of ecosystem health, zoonotic risks, and anthropogenic pressures. Systematic necropsies provide valuable insights into mortality patterns, yet regionally structured evaluations in Central Europe remain scarce.

**Methods:**

A total of 2,118 complete necropsy reports of wild mammals submitted to public veterinary investigation authorities in southern Germany between 2019 and 2023 were retrospectively analyzed. Data were categorized according to species, age, sex, season, and cause of death. Mortality causes were classified using the WHO ICD-11 system to enable standardized reporting and international comparability.

**Results:**

The most frequently examined species were European hare *(Lepus europaeus)*, red fox *(Vulpes vulpes)*, roe deer *(Capreolus capreolus)* and wild boar *(Sus scrofa)*. Infectious diseases were the leading cause of death, with tularemia and European brown hare syndrome predominantly affecting European hares, rabbit hemorrhagic disease occurring mainly in European rabbits and canine distemper virus representing the major infectious cause of death in red foxes, often showing distinct regional clustering. Trauma was the second most common cause of death, with road traffic collisions as the predominant factor. A pronounced seasonal mortality peak was observed in spring, particularly among adults.

**Discussion:**

The present study highlights the multifactorial nature of wildlife mortality in southern Germany, with anthropogenic drivers playing a central role. It represents the first comprehensive regional overview of wildlife mortality in Germany based on complete necropsy datasets from all public veterinary investigation authorities in southern Germany. The ICD-11 classification proved useful for structuring mortality data and supports international comparability. These findings underline the value of necropsy-based surveillance as an important tool in One Health, informing conservation planning, wildlife management and public health risk assessment.

## 1 Introduction

Understanding the causes of death in wild mammals is essential for assessing ecological pressures and anthropogenic impacts across regions (1).

Wildlife health and well-being are compromised by both natural and human related stressors. Natural factors include limited water and food resources, infectious diseases, predation, parasitism and accidental trauma such as falls or drowning (2). In contrast, anthropogenic pressures such as vehicle collisions, poisoning, poaching, and habitat destruction have been identified as major contributors to wildlife mortality in Europe (1). Some species increasingly exploit human dominated landscapes, leading to frequent interactions, as observed in urban foxes and hedgehogs in Germany (3).

Urbanization and land use changes exacerbate these pressures by increasing traffic density, expanding infrastructure and fragmenting habitats. Currently, around 80% of the European population resides in urban or suburban areas, a proportion expected to rise further worldwide (4). In Germany, settlement density and traffic volumes have risen markedly over recent decades (5). Despite the recognized relevance of these developments, their influence on wildlife mortality across species remains insufficiently understood at the regional scale.

Germany hosts 3.5% of the world’s known animal species, including 104 mammalian species (6). According to the German Hunting Association’s “*Tierfund-Kataster*,” around 250.000 wildlife-vehicle collisions occur annually in Germany, predominantly involving large mammals such as deer and wild boar (7).

The determination of wildlife mortality causes is routinely conducted as part of official monitoring programs in Baden-Wuerttemberg and Bavaria. Organizations such as the Baden-Wuerttemberg Chemical and Veterinary Investigation Offices, the State Veterinary Investigation Office in Aulendorf, the Bavarian State Office for Health and Food Safety, the Bavarian Animal Health Service, and the Institute of Veterinary Pathology at the LMU Munich perform necropsies to document causes of death in wild mammals. The submission of carcasses for examination is not mandatory and depends on opportunistic reporting and voluntary submission by authorities, hunters, wildlife managers or private individuals. Consequently, there is no comprehensive or standardized recording of all deceased wild animals, and the examined cases represent only a subset of overall wildlife mortality. For the present study, all available necropsy reports within the study period were screened and only cases with complete diagnostic documentation were included in the final analysis. While some findings usually are published in regional wildlife reports or the German Veterinary Journal (*Deutsches Tieraerzteblatt)*, primarily for notifiable disease, a regional analysis of anthropogenic and non-anthropogenic causes of cross-species mammalian wildlife mortality in southern Germany remains absent from the current literature. Notably, international studies often focus on a single species rather than presenting systematic, multi-species assessment.

This retrospective study investigates the primary causes of death in wild mammals in southern Germany from 2019 to 2023, incorporating both biological and anthropogenic factors. The analysis is based on necropsy records from all public veterinary investigation authorities in Baden- Wuerttemberg and Bavaria. By examining the interplay between infectious and non-infectious events, the study adopts a One Health perspective to highlight the interconnectedness of human, animal, and environmental health. Causes of death were coded using the ICD-11 (International Classification of Diseases) system, developed by the World Health Organization (WHO). The system was originally designed for human medicine to provide a harmonized classification of diseases and causes of death and enable international comparisons. For the present study, the ICD-11 scheme was adapted to allow a uniform classification of wild mammal mortality. Where necessary, the main diagnostic categories were modified to account for the specific characteristics of veterinary cases. This approach has previously been applied by Marchetti et al. in a comparable study conducted in Italy (8).

Due to the lack of standardized statistical documentation of wildlife mortality in southern Germany, the present study addresses a relevant knowledge gap. The results aim to support evidence-based conservation strategies and improve the understanding of environmental and anthropogenic risks to wildlife and human health.

## 2 Materials and methods

Wildlife necropsy reports were obtained over a 5-years period from 2019 to 2023 from all official diagnostic institutions in Southern Germany, defined here as the federal states of Baden-Wuerttemberg and Bavaria. The region has a temperate climate with mean annual temperatures of approximately 8–10 °C and moderate precipitation, increasing toward mountainous areas (9). Elevations range from lowland river valleys to the Black Forest and the Bavarian Alps (up to 2,962 m) (10, 11). Land use is dominated by agriculture (approximately 45–46%) and forest (35–38%), while settlement and transport infrastructure account for around 12–15% of the area (12, 13). This heterogeneous landscape creates a mosaic of natural and anthropogenically influenced habitats.

Participating institutions were the Baden-Wuerttemberg Chemical and Veterinary Investigation Offices, the State Veterinary Investigation Office in Aulendorf, the Bavarian State Office for Health and Food Safety, the Bavarian Animal Health Service, and the Institute of Veterinary Pathology at the LMU Munich.

Necropsies were performed by veterinary pathologists according to internal standard operating procedures (SOPs).

Only reports of wild mammals that died due to natural causes or unintentional human impacts were included in this study. Animals that were legally hunted, including animals killed due to disease control or human dispatch, as well as euthanized animals, were excluded. Specimens submitted by hunters or authorities within official surveillance programs (e.g., rabies monitoring) were included only if death was not caused by hunting or active killing. Domesticated animals (i.e., pets such as dogs and cats) were excluded. Only complete necropsy reports with sufficient diagnostic information for cause-of-death assessment were considered. Cases with incomplete documentation were excluded from analysis.

For each animal, the following information was recorded and analyzed in this study: Signalment including sex, nutritional status, age group (if identifiable), degree of autolysis, date and location of discovery using the postal code (if available), date of necropsy, necropsy findings including bacteriological, virological, parasitological and toxicological laboratory results (if performed), cause of death, and comorbidities.

Causes of death were categorized based on etiology into four main groups to ensure meaningful group sizes and maintain etiologic consistency: (I) Infectious/inflammatory disease, (II) trauma, (III) Metabolic- and toxic diseases and (IV) Other causes including congenital and developmental abnormalities, circulatory diseases and neoplasia. This classification was derived from the diagnostic criteria documented in the necropsy reports and ensure consistent categorization across cases.

For a comparative approach, causes of death were additionally coded using the International Classification of Diseases, 11th Revision (ICD-11) of the World Health Organization (WHO) (14).

Statistical analysis was conducted using Microsoft Excel (Version 16.91) and GraphPad Prism (Version 5.04).

### 2.1 Data source and data collection

The data were derived from necropsy reports prepared by the official diagnostic laboratories.

The reports followed a standardized structure, including:

- Anamnesis form containing the submitter’s contact details, time, species of the submitted mammal, and additional information such as age, if provided by the submitter.
- Macroscopic findings and histopathological examination results.
- Laboratory diagnostic analyses including bacteriological, virological, and parasitological examinations.
- Interpretation of findings including the cause of death.

### 2.2 Data processing and statistical analysis

Data were recorded and processed using a custom designed Microsoft Excel file (version 16.91), structured according to the format of the necropsy reports. Furthermore, the ICD-11 scheme was imported into Excel via csv (comma-separated values) format and integrated into the table for linkage through a dropdown menu, facilitating the generation of the corresponding ICD-11 code.

Both species and causes of death were systematically categorized. In cases of trauma due to bite injuries, further differentiation into wild predator- related, domestic animal- related or intraspecific aggression was based on available case history and lesion morphology as documented in the necropsy reports. When the origin of the bite could not be reliably inferred, cases were classified as bite wounds of undetermined origin.

Amyloidosis was assigned to the metabolic/toxic category as it represents a systemic protein misfolding and deposition disorder leading to organ dysfunction, irrespective of a potentially underlying chronic inflammatory trigger.

Age was classified as neonate, juvenile, adult or geriatric. When available, exact age information was provided by the submitter, for example for animals that died at rehabilitation or care facilities. In all other cases age was estimated by the pathologist based on morphological or reproductive characteristics. Classification was guided by biological indicators such as dependence on maternal care, sexual maturity, and signs of senescence like severe tooth wear.

Nutritional status was classified as good, moderate, or cachectic based on information given in the necropsy reports. Emaciation was assigned to the metabolic/ toxic category when no evidence of infectious disease, trauma, congenital anomaly, or other identifiable primary pathology was documented. In this retrospective setting, acute food deprivation or environmental nutrient deficiency could not be reliably distinguished and was therefore not recorded as a separate category.

The analysis included descriptive statistics, frequency distributions, and correlation analyses between various parameters using chi-squared tests based on cross tables where the case numbers of the most common investigated species (n > 50) were assigned to the respective diagnosed cause of death categories as defined above. Data were recorded and processed using Microsoft Excel (Version 16.91), and GraphPad Prism (Version 5.04) was used for evaluation and statistical analysis. A *p*-value <0.05 was considered statistically significant. Possible correlations between species and main cause of death were investigated, as well as potential correlations between main cause of death and location of discovery, age, sex, and nutritional status.

## 3 Results

### 3.1 Geogaphical and seasonal distribution

**Figure 1:**
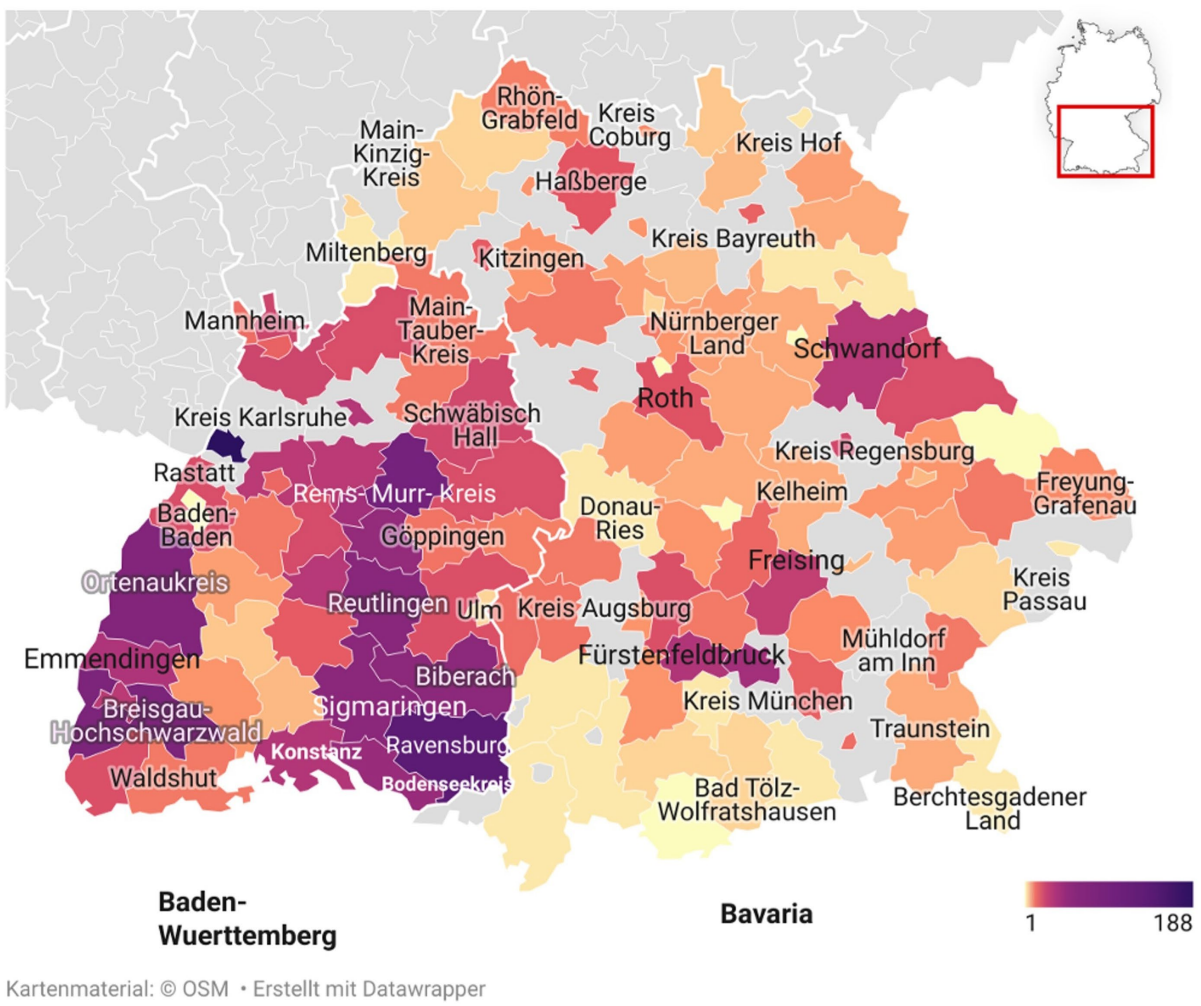
Distribution of reported wildlife carcass findings submitted for necropsy in southern Germany (2019–2023). The color scale indicates the number of cases per discovery site based on necropsy reports. *Map data: © OpenStreetMap contributors; visualization created using Datawrapper.

Between 2019 and 2023, carcasses of 2,118 wild mammals were investigated originating from 118 of 140 different administrative districts within the federal state of Baden- Wuerttemberg (66%; *n* = 1391/2118) and Bavaria (31%; *n* = 653/2118). In 3% (*n* = 74/2118) of the cases, no precise geographic assignment was possible due to insufficient information in the submission form. The regional distribution of the examined animals in southern Germany is shown in Figure 1. A peak in the number of submissions was noted in 2019 with 25% of all cases (*n* = 536/2118). During the following years 2020 (19%; *n* = 409/2118), 2021 (20%; *n* = 420/2118) and 2022 (20%; *n* = 426/2118), a balanced number of cases were investigated. In 2023, the lowest number of cases were reported with 15% (*n* = 327/2118). The highest percentage of cases occurred, regardless of cause, during spring (meteorological definition: March to May; 29%; *n* = 619/2118), followed by autumn (defined as September to November; 28%; *n* = 601/2118), winter (defined as December to February; 21%; *n* = 452/2118), and summer (defined as June to August; 21%; *n* = 446/2118).

**Figure 2:**
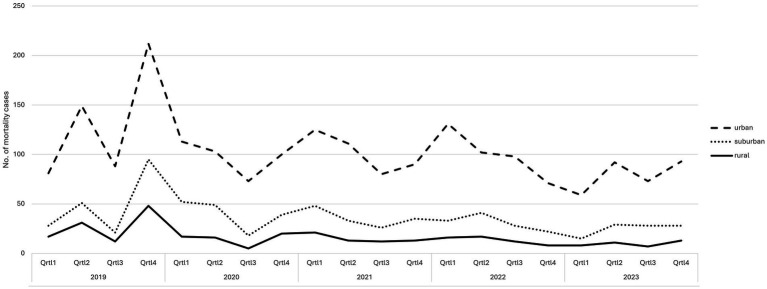
Quarterly (Q) figures on reported mortality cases (absolute numbers) in wild mammals based on veterinary necropsy between 2019 to 2023, differentiated by place of recovery (rural, suburban, urban).

The temporal distribution of mortality cases across different types of discovery sites over the five-year study period is shown in Figure 2. Sites were classified according to settlement type, following the definition of the Federal Statistical Office, based on population density: rural (<150 inhabitants/km^2^), suburban (150–300 inhabitants/km^2^), and urban (>300 inhabitants/km^2^) areas (15). A peak was observed for all region types in the last quarter of 2019 with urban areas showing the highest absolute numbers (6%; *n* = 117/2044). Following this peak, a general decline in mortality cases was evident across all region types, with some fluctuations throughout the observation period.

During the late first quarter of 2020 to early second quarter of 2020, and last quarter of 2020 to early first quarter of 2021 a marked decrease in the number of carcasses submitted for necropsy was observed, particularly in urban and suburban regions. Rural areas also showed a decline in submissions, albeit less pronounced.

Throughout the whole study period, urban regions (65%; *n* = 1325/2044) consistently exhibited higher absolute numbers of mortality cases compared to suburban (20%; *n* = 402/2044) and rural regions (15%; *n* = 317/2044). However, the differences between region types tend to decrease over time.

### 3.2 Signalment

The animals examined represent 35 different species ranging from Rodentia, Carnivora, Ungulata, Eulipotyphla, and Chiroptera. A complete overview including case numbers is given in Table 1.

**Table 1:** Overview of wild mammal species with taxonomic classification and case numbers.

No	Order	Family	Species (lat.)	Cases
1	Rodentia	Leporidae	European hare (*Lepus europaeus)*	659
2			European rabbit (*Oryctolagus cuniculus)*	56
3		Castoridae	Eurasian beaver (*Castor fiber)*	61
4		Sciuridae	Eurasian red squirrel (*Sciurus vulgaris*)	46
5			Dormouse (general) (*Gliridae* spp.*)*	1
6			Edible dormouse (*Glis glis)*	1
7		Muridae	Brown rat (*Rattus norvegicus)*	11
8			House mouse (*Mus musculus*)	4
9		Cricetidae	Coypu (*Myocastor coypus*)	3
10			Common vole (*Microtus arvalis*)	2
11	Carnivora	Canidae	Red fox (*Vulpes vulpes*)	393
12			Gray wolf (*Canis lupus*)	1
13		Mustelidae	Pine−/ Stone marten (*Martes martes/ Martes foina*)	70
14			European badger (*Meles meles*)	34
15			Eurasian otter (*Lutra lutra*)	3
16			European polecat (*Mustela putorius*)	2
17			Racoon dog (*Myctereutes procyonoides*)	2
18			Least weasel (*Mustela nivalis*)	2
19			American mink (*Neogale vison*)	1
20			Stoat/ Ermine (*Mustela erminea*)	1
21		Felidae	European wildcat (*Felis silvestris*)	92
22			Eurasian lynx (*Lynx lynx*)	1
23			Racoon (*Procyon lotor*)	16
24	Ungulata	Cervidae	Roe deer (*Capreolus capreolus*)	267
25			Red deer (*Cervus elaphus*)	13
26		Suidae	Wild boar (*Sus scrofa*)	222
27		Bovidae	Chamois (*Rupicapra rupicapra*)	3
28	Eulipotyphla	Erinaceidae	European hedgehog (*Erinaceus europaeus*)	99
29		Talpidae	European mole (*Talpa europaea*)	7
30		Soricidae	Shrew (general) (*Soricidae spp*)	2
31	Chiroptera	Vespertilionidae	Common pipistrelle (*Pipistrellus pipistrellus*)	20
32			Bat (unspecified) (*Vespertilionidae* spp.*)*	15
33			Geoffroy’s bat (*Myotis emarginatus*)	6
34			Fringed long- eared bat (*Myotis nattereri*)	1
35			Daubenton’s bat (*Myotis daubentonii*)	1

The most frequently examined species was the European hare (*Lepus europaeus*, 31%), followed by the red fox (*Vulpes vulpes*, 19%), the roe deer (*Capreolus capreolus*, 13%), and the wild boar (*Sus scrofa*, 10%).

Sex was determined for each animal and was overall distributed equally, with 48% (*n* = 1010/2118) males and 44% (*n* = 929/2118) females. In 8% of the cases (*n* = 174/2118), no sex information was available.

In terms of age, the adult group dominated (55%; *n* = 1162/2118), followed by neonates (13%; *n* = 272/2118), juveniles (11%; *n* = 242/2118), and geriatric animals (6%; *n* = 116/2118). The ratio between geriatric plus adult to juvenile plus neonate animals was 2.5:1. No information on the age was available in 15% (*n* = 326/2118).

The documented nutritional status was good to very good in 35% (*n* = 746/2118), while 27% (*n* = 580/2118) were moderately nourished and 25% (*n* = 523/2118) cachectic. Cachexia showed a significant statistical association with metabolic/toxic causes of death (χ^2^ = 112.3; df = 4; *p* < 0.0001). However, many cachectic animals presented with additional pathological findings. Among cachectic individuals, 78% (*n* = 408/523) had coexisting infectious diseases. In these cases, if the infectious disease was considered the primary lethal process, the case was classified under infectious/inflammatory causes of death, and cachexia was interpreted as secondary. Conversely, emaciation due to unclear origin, dietary deficiency, or functional impairment affecting food intake was classified under metabolic/toxic causes. In 6% of cachectic animals (*n* = 29/523), cachexia was considered the primary cause of death in the absence of any identifiable underlying pathology.

### 3.3 Causes of death

In 98% (*n* = 2083/2118) of the included cases a primary cause of death could be determined based on the available pathological findings, whereas advanced autolysis precluded accurate assessment in the remaining 2% (*n* = 35/2118). Analysis of the necropsy data revealed a distribution of 70% (*n* = 1479/2118) accounting for non-traumatic and 30% (*n* = 639/2118) for traumatic causes of death.

Group I: Infectious and/or inflammatory causes of death accounted for 62% of all examined cases (*n* = 1323/2118). Within this group, bacterial infections were most frequent (39%; *n* = 516/1323), followed by viral infections (23%; *n* = 303/1323), and parasitic diseases (22%; *n* = 293/1323). Furthermore, there were nine cases of mycotic infections (1%; *n* = 9/1323). This category includes infectious diseases as the primary cause of death as well as severe infections that contributed substantially to death.

Infectious diseases predominantly affected European hares with *Francisella tularensis* identified as the primary cause in 34% of cases (*n* = 227/516). European brown hare syndrome (EBHS), caused by European brown hare syndrome virus (EBHSV), a calicivirus of the genus lagovirus, was diagnosed in 14% of cases in hares (*n* = 93/516). In European rabbits, rabbit hemorrhagic disease virus (RHDV*)*, another Lagovirus, was the most frequently detected infectious agent and was identified in 6% of cases (*n* = 33/516).

In red foxes, canine distemper virus represented the leading infectious cause of death (37%; *n* = 146/393). Ectoparasitic infestations, particularly with *Sarcoptes scabiei* (8%; *n* = 30/393), were frequently recorded but were not uniformly lethal and often acted as contributing factor through chronic debilitation and secondary infections. Respiratory diseases (13%; *n* = 36/267) were increasingly observed in roe deer, mostly associated with primary endoparasite infections. Table 2 summarizes the most frequently detected infectious agents in wildlife species with more than 50 examined individuals.

**Table 2:** Most frequently detected pathogens in selected wild mammal species with more than 50 cases, including prevalence (%) and case numbers (n).

Species (lat.)	Pathogens (grouped by type)	Prevalence
European hare *(Lepus europaeus)*	Bacterial: *Francisella tularensis* *Yersinia* spp.	34% (*n* = 227/659) 7% (*n* = 47/659)
	Viral: European Brown Hare Syndrome Virus	15% (*n* = 97/659)
Red fox (*Vulpes vulpes*)	Viral: Canine Distemper Virus	37% (*n* = 146/393)
	Parasitic: *Sarcoptes scabiei* *Crenosoma vulpis* *Echinococcus* spp.	8% (*n* = 30/393) 1% (*n* = 5/393) 1% (*n* = 5/393)
Roe deer (*Capreolus capreolus*)	Parasitic: *Strongylidae/ Trichostrongylidae* *Dictyocaulus viviparus*	16% (*n* = 45/276) 7% (*n* = 19/276)
	Bacterial: *Clostridium* spp.	9% (*n* = 25/276)
	Fungal: *Aspergillus* spp.	2% (*n* = 5/276)
Wild boar (*Sus scrofa*)	Parasitic: *Metastrongylus* spp.	15% (*n* = 33/222)
	Bacterial: *Pasteurella multocida*	5% (*n* = 10/222)
	Viral: Suid Herpesvirus-1 Porcine Circovirus- 2	1% (*n* = 2/222) 1% (*n* = 2/222)
European hedgehog (*Erinaceus europaeus*)	Parasitic: *Crenosoma striatum*	27% (*n* = 27/99)
	Bacterial: *Salmonella* spp.	21% (*n* = 21/99)
European wildcat (*Felis silvestris*)	Bacterial: *Mycoplasma* spp.	1% (*n* = 1/92)
	Viral: Feline Leukemia Virus	1% (*n* = 1/92)
Pine- and Stone marten (*Martes martes/ Martes foina*)	Viral: Canine Distemper Virus	19% (*n* = 13/70)
Eurasian beaver (*Castor fiber*)	Parasitic: *Echinococcus* spp.	5% (*n* = 3/61)
European rabbit (*Oryctolagus cuniculus*)	Viral: Rabbit Hemorrhagic Disease Virus Myxoma Virus	59% (*n* = 33/56) 2% (*n* = 1/56)

Group II: Traumatic causes of death accounted for 30% (*n* = 640/2118) of all examined cases. Within this group, blunt force trauma predominated (76%, *n* = 490/640), followed by sharp force trauma (18%, *n* = 115/640), and other types of trauma (6%, *n* = 35/640). Among blunt force injuries, 44% (*n* = 215/490) were of anthropogenic origin, predominantly resulting from traffic collisions (43%, *n* = 210/490). These cases were diagnosed either based on the case history or inferred from characteristic injury patterns consistent with vehicular impact. The remaining 56% (*n* = 275/490) had an undetermined origin. Wild boars, European wildcats, European beavers and martens were particularly affected, with traffic collisions being the leading cause of death. Slightly more males (46%; *n* = 97/210) than females (43%; *n* = 90/210) were involved in these incidents, while sex was not specified in 11% (*n* = 23/210).

Sharp forced trauma was mainly linked to bite injuries caused by wild predators (17%, *n* = 19/115), domestic animals (15%, *n* = 17/115), and intraspecific aggression (2%, *n* = 2/115), while in 30% (*n* = 34/115) the source of the bite could not be identified. Anthropogenic sharp trauma included hunting-related gunshot wounds (26%, *n* = 30/115), and two cases caused by mowing equipment; 10% (*n* = 11/115) of sharp force trauma were of unclear origin.

Other trauma involved organ injuries caused by mechanical force or foreign bodies (31%, *n* = 11/35), trauma-related complications such as brain abscesses following traumatic antler fracture (46%, *n* = 16/35), barotrauma associated with wind turbine facilities (11%, *n* = 4/35), and drowning/asphyxiation (11%, *n* = 4/35). Injuries classified under mechanical force comprised for example splenic rupture with subsequent circulatory failure or rupture of the left atrium with cardiac tamponade. These events were attributed to mechanical trauma when no underlying pathological condition (e.g., neoplasia) contributed to organ failure. Such cases were not grouped under blunt trauma because death resulted primarily from internal organ failure rather than the external impact itself.

**Figure 3:**
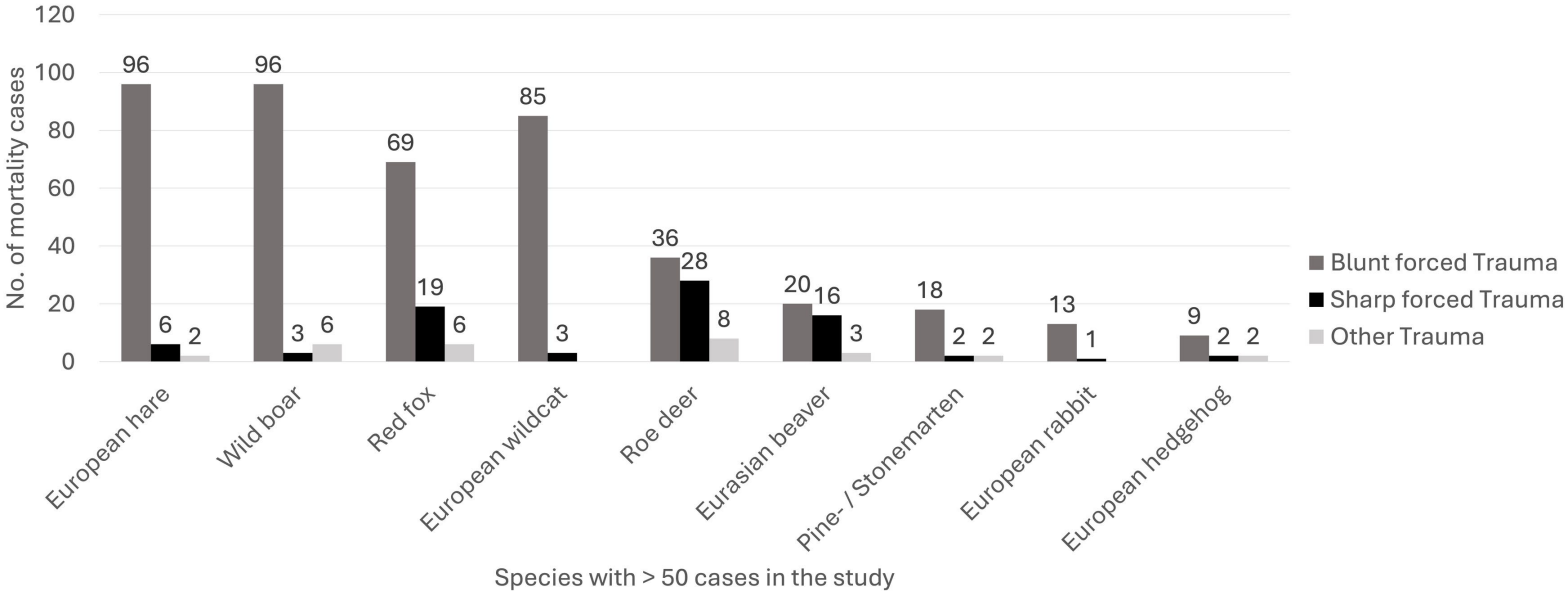
Stacked bar chart depicting the distribution of traumatic causes of death by trauma type in the 10 most frequently represented wildlife species (> 50 cases) examined in Southern Germany between 2019 and 2023.

Overall, 42% (*n* = 270/640) of trauma related deaths were attributed to anthropogenic causes. Traumatic events occurred more frequently in spring (27%; *n* = 176/640) and summer (28%; *n* = 178/640) compared to autumn (24%; *n* = 155/640) and winter (20%; *n* = 131/640). Figure 3 presents the distribution of traumatic causes of death by trauma type in the nine most frequently represented wildlife species (> 50 cases).

Group III: Metabolic- and toxic causes of death accounted for 4% of all examined cases (*n* = 77/2118) and primarily including severe emaciation. Within this group, amyloidosis was diagnosed in 14% of cases (*n* = 11/77), predominantly in pine- and stone martens, affecting the liver, spleen and kidneys. Toxic causes represented 10% of metabolic and toxic cases (*n* = 8/77) and included exposure to coumarin and carbofuran. The cases affected multiple species, including two rats (coumarin), one roe deer (ivy), two martens (one coumarin, one unknown), one badger (cumatetralyl), one red deer (unknown), and one fox (carbofuran).

Group IV: Other causes of death including congenital and developmental abnormalities, circulatory disorders and neoplasia, accounted for 2% of all examined cases (*n* = 43/2118). Within this group, circulatory disorders represented 51% of cases (*n* = 22/43) and were defined as diagnosis of exclusion, assigned when death was attributed to cardiovascular failure in the absence of specific gross or histopathological lesions. Neoplasia accounted for 28% of cases in this category (*n* = 12/43) and occurred predominantly in adult females (67%; *n* = 8/12). Congenital anomalies and developmental malformations comprised 21% of group IV cases (*n* = 9/43) and included hydrocephalus, severe dental wear and various organ malformations.

Supplementary Table S1 shows an overview of non-traumatic and traumatic causes of death in absolute numbers and percentages by cause of death, sex, age, and season (irrespective of species) for the submitted cases included in this study. Supplementary Table S2 provides additional details on anthropogenic traumas following the same pattern. The Chi-squared test revealed a highly significant association between species (species represented by >50 cases) and causes of death (x^2^ = 97.95; df = 12; *p* < 0.0001), indicating that mortality patterns varied considerably between taxa. Infectious and inflammatory diseases were the predominant causes of death in European hares, European Hedgehogs, roe deer, red foxes and European rabbits. In contrast, traumatic causes of death, including traffic accidents and other forms of mechanical trauma, were most frequently in wild boars, European wildcats, Eurasian beavers, pine and stone martens.

A significant association was also found between causes of death and both age (x^2^ = 40.41; df = 12; *p* < 0.0001) and season (x^2^ = 58.92; df = 12; *p* < 0.0001), indicating age- and season specific differences in the distribution of mortality causes across species. In contrast, no significant association was detected between sex and cause of death (x^2^ = 5.918; df = 4; *p* = 0.2054). Independent of cause of death-category, most carcasses were recorded in spring, particularly among European rabbits, wild boars, European wildcats, roe deer, Eurasian beavers, European hares and red foxes. In pine- and stone martens, the number of recorded deaths peaked during summer. Across all species, adults represented the majority of examined individuals, except in European hedgehogs, where juvenile and adult cases occurred in nearly equal proportions. Among red foxes, a comparatively high proportion of individuals were classified as geriatric.

Although a higher proportion of deaths due to anthropogenic causes were recorded in Baden-Wuerttemberg (21%; *n* = 297/2118) compared to Bavaria (15%; *n* = 99/2118), no significant association was found (x^2^ = 5.849; df = 2; *p* = 0.0537).

### 3.4 Prevalence of secondary diagnosis

In 39% of the cases (*n* = 835/2118) additional secondary diagnoses were recorded alongside the primary causes of death. Parasitic infection represented the most frequent secondary finding (68%; *n* = 564/835), commonly observed in animals showing poor general condition or other concurrent health disorders. European hares (28%; *n* = 156/564) were particularly affected by intestinal coccidiosis and lungworm infestations frequently associated with severe emaciation. A similar pattern was observed in roe deer (21%; *n* = 119/564) where infections with coccidia *(Eimeria* sp.*)*, strongylid nematodes, and trichostrongylid nematodes were linked to emaciation and anemia.

### 3.5 Zoonoses

Potentially zoonotic pathogens were detected in 15% of all recorded cases (*n* = 315/2118). Within this group the most common were infections with *Francisella tularensis* (73%; *n* = 230/315), followed by *Salmonella* spp. (16%; *n* = 50/315), and parasitic pathogens such as *Echinococcus multilocularis* (4%; *n* = 12/315) and *Cryptosporidium* spp. (4%; *n* = 12/315). European hares were affected disproportionately often (75%; *n* = 235/315). In 84% of the cases (*n* = 264/315), the infections were directly related to the cause of death, while documented as secondary diagnoses in 16% (*n* = 51/315). Regional clusters of zoonotic pathogens were found in the urban areas of Baden-Wuerttemberg (55%; *n* = 174/315). Table 3 shows an overview of all diagnosed zoonoses in this study.

**Table 3:** Overview of zoonotic pathogens including total case numbers, affected species and classification as primary or secondary finding.

Pathogen	Case numbers	Main species	Cause of death in absolute numbers	Incidental finding in absolute numbers
*Francisella tularensis*	230	European hare	219	11
*Salmonella* spp.	50	European hedgehog	34	16
*Echinococcus multilocularis*	12	Red fox	2*	11
*Cryptosporidium* spp.	12	European hedgehog	2	10
*Toxoplasma gondii*	4	European hare, Eurasian beaver, Eurasian red squirrel, Roe deer	3	1
*Anaplasma phagocytophilum*	2	Roe deer	0	2
*Listeria monocytogenes*	2	Roe deer, Eurasian beaver	2	0
*Leprospira* spp.	2	Roe deer, European hedgehog	2	0
*Brucella* spp.	1	European hare	1	0

*The two fatal cases of Echinococcus multilocularis infection occurred in Eurasian beavers. Infections detected in red foxes represented incidental findings without lethal outcome.

In addition, 30% (*n* = 626/2118), primarily red foxes (61%; *n* = 381/626), were tested for rabies as part of routine surveillance with negative results. In 2021, 0.3% (*n* = 6/2118) were also tested for SARS-CoV-2, including a bat, European wildcats, a European hedgehog, and an American mink. All animals were tested negative.

### 3.6 ICD-11

The WHO classification scheme (ICD-11) was used for the standardized categorization of causes of death. The cases could be assigned to the superordinate ICD-11 chapters, which enables a systematic classification into disease-related main groups.

The most frequent main categories concerned Chapter 1 (“Certain infectious or parasitic diseases”) and Chapter 23 (“External causes of morbidity or mortality”). Table 4 shows an overview of the distribution of cases according to ICD-11 chapters as well as an example of coding according to etiology.

**Table 4:** Classification of recorded mortality cases according to ICD-11 chapters and codes including genesis and percentage distribution.

ICD-11 chapter	Code range	Description	Cases	%	Genesis	ICD-11 code	Keyword	Cases	%
1 Infectious or parasitic diseases	1A00–1H0Z	Infectious/ inflammatory Diseases	1,323	62	bacterial	1C4Z	Unspecified bacterial disease	516	39
					Viral	1E1Z	Unspecified viral disease	303	23
					Parasitic	1G2Z	Unspecified parasitic disease	293	22
					Mycotic	1F20. Z	Aspergillosis	9	0.6
2 Neoplasm	2A00–2F9Z	Neoplastic Diseases	12	0.6	Hematopoietic or lymphoid tissue	2B3Z	Neoplasms of hematopoietic or lymphoid tissue, unspecified	7	58
					Remaining tissue	2F9Z	Neoplasms of unknown behavior of unspecified site	5	42
5 Endocrine, nutritional or metabolic diseases	5A00–4D46	Metabolic/ Toxic Diseases	77	4	Metabolic	5D2Z	Metabolic disorders, unspecified	69	90
					Toxic	NE60	Harmful effects of drugs, medicaments or biological substances, not elsewhere classified	8	10
11 Disease of the circulatory system	BA00–BE2Z	Cardiovascular Disease	22	1	Circulation	BE2Z	Diseases of the circulatory system, unspecified	22	100
20 Developmental anomalies	LA00–LD9Z	Congenital diseases/ Malformations	9	0.4	Malformations/ Anomalies	LD9Z	Developmental anomalies, unspecified	9	100
23 External causes of morbidity and mortality	PA00–PL2Z	Trauma (e.g., traffic collisions, predation)	640	30	Unintentional causes	PB6Z	Unspecified unintentional cause of morbidity or mortality	640	100

## 4 Discussion

This retrospective evaluation of 2,118 necropsy reports from wild mammals in southern Germany (2019–2023) provides a comprehensive overview of documented regional wildlife mortality.

The dataset does not represent all wildlife deaths but includes only animals submitted to public veterinary investigation authorities for postmortem examination. Necropsies are performed after carcass discovery or within targeted monitoring and notifiable disease surveillance programs. There is no legal obligation to submit wildlife carcasses and documentation remains decentralized.

As a result, the dataset is affected by inherent selection bias related by detectability, species size, public awareness, and perceived disease relevance. Large and conspicuous species are therefore overrepresented. This bias reflects the structure of the existing wildlife surveillance system rather than methodological limitation of the present study. By compiling necropsy data from all southern German public veterinary investigation authorities, this study provides the first integrated regional analysis of documented mammalian wildlife mortality in Germany. The result must be interpreted in the context in which the data were generated. Within this framework, infectious diseases and trauma constituted the dominant causes of death, with anthropogenic factors playing a central role. The application of ICD-11 enabled structured categorization of mortality causes and facilitated comparison with international datasets. Seasonal, demographic and spatial patterns observed in this study reflect the interaction between ecological processes, pathogen dynamics and human activity.

The most frequently examined species were European hare, red fox, roe deer and wild boar, which are ecologically prominent in southern Germany and commonly reported in European wildlife health surveys (16). Other causes of death including metabolic- and toxic conditions, congenital and development abnormalities, circulatory diseases and neoplasia occurred only sporadically and were not analyzed further. Accordingly, the following section focuses on infectious and traumatic mortality as the most epidemiologically relevant categories.

### 4.1 Seasonal and demographic patterns

Analyses of seasonal and demographic structures support a more precise interpretation of mortality patterns and helps to identify potential ecological drivers as well as sampling biases. Adult animals predominated among submitted cases. This reflects a detection and submission bias rather than an increased intrinsic susceptibility. Adult carcasses are more frequently encountered and reported, particularly along roads or in accessible areas. In contrast, juvenile animals often die in secluded habitats, where carcasses are rapidly removed by scavengers or decomposed and therefore remain undetected (17–19). In addition, adult individuals affected by disease or chronic conditions are more likely to be noticed due to altered behavior, reduced mobility or proximity to human infrastructure. This further contributes to their overrepresentation in necropsy-based datasets (20). A pronounced seasonal peak in mortality was observed in spring, particularly among adult red foxes, roe deer and wild boars. These findings can be attributed to a combination of ecological, behavioral and physiological factors. During mating and dispersal, these species exhibit increased activity, territorial defense, and risk-taking behaviors, which may elevate intra- and interspecific conflicts, predator encounters, and road crossings (21, 22). Seasonal stress may further contribute to mortality. After winter, many individuals remain in reduced body condition, while rising temperatures and frequent population contacts facilitate pathogen transmission. Together, these factors explain the concurrent increase in both infectious and traumatic deaths observed in spring (23).

### 4.2 Regional and methodological aspects

The predominance of infectious causes in this study reflects not only ecological conditions but also regional and methodological factors. Southern Germany is characterized by comparatively dense and well-structured wildlife health surveillance systems. Baden-Wuerttemberg operates a state-wide necropsy program free of charge, resulting in high submission rates. Bavaria maintains a similarly dense surveillance network, although submissions are not uniformly free of charge (24). These differences influence both case numbers and the scope of diagnostic investigations and must be considered when comparing datasets at the national level.

Regional environment represents an additional driver of wildlife mortality. In southern Germany, the close spatial intermixing of settlements, agriculture land and natural habitats creates a highly fragmented landscape (25). This fragmentation enhances edge effects and expands contact zones between humans, domestic animals and wildlife. Higher land use intensity is therefore associated with elevated encounter rates across species boundaries, facilitating pathogen transmission and secondary infections even in predominantly rural regions. In addition, increased population density and habitat fragmentation may intensify intraspecific territorial competition, leading to aggressive interactions and trauma-related mortality.

### 4.3 Urban mortality in 2019

An unexpectedly high number of wildlife fatalities in urban areas was recorded in 2019, primarily related to traffic collisions. Several factors may explain this observation. Favorable climatic conditions in preceding years, including mild winters, supported high population densities in species such as roe deer, wild boar, and European hare (26, 27). Reduced food availability in rural areas, such as after the drought in 2018, may have compelled wild mammals to shift toward suburban and urban habitats, where resources were more accessible. This is supported by findings that the relative abundance of certain hunted species, such as red foxes in Germany, is elevated in residential yards, indicating increased urban foraging behavior under resource stress (28, 29). In addition, traffic volumes were high in 2019, in contrast to the subsequent pandemic-related restrictions. Infrastructure development (e.g., road construction) may have further influenced wildlife movement and collision risk (30). According to the German Hunting Association around 237,000 wildlife vehicle collisions were recorded nationwide in the 2019/20 hunting year (31). Although subsequent years (2020/21–2022/23) showed slightly lower totals, collision numbers remained high, reflecting fluctuations in traffic intensity and reporting practices.

Police accident statistics from the Federal Statistical Office of Germany (Destatis) indicate that wildlife collisions represent a substantial subset of traffic accidents involving personal injury, even though only a fraction of wildlife vehicle collision are captured in official police records (32, 33). Integrating data on wildlife collisions from both ecological and traffic statistics thus provides a more robust understanding of the temporal peaks observed in urban wildlife mortalities, such as the one documented in 2019.

### 4.4 Infectious causes of death

Infectious and inflammatory diseases constituted the most frequent primary causes of death in this study and showed clear species-specific patterns. Tularemia was particularly prevalent in lagomorphs, confirming the endemic status of *Francisella tularensis* in southern Germany (34). Long-term monitoring by the Bavarian Hunting Association has shown persistently high positivity rates of around 30% in European brown hares since 2019 (35). A considerable proportion of the European brown hare cases in the present study originated from this project. Nothdurfter et al. documented repeated detections of *Francisella tularensis* infections in brown hares in Baden Wuerttemberg over a 10-year period. The recurrent detection of *F*. *tularensis* underscores the role of the brown hare as an important reservoir host. It also highlights the zoonotic exposure risk for hunters, farmers, and veterinarians. Human surveillance data support this concern. In Baden-Wuerttemberg, 152 human tularemia cases, including five fatalities, were reported between 2012 and 2022, with annual case numbers ranging from 3 to 34 and a peak in 2021. Arthropod bites, particularly from ticks, accounted for 45% of exposures, whereas direct contact with wildlife was reported in fewer than 15% of cases. Baden-Wuerttemberg consistently ranks among the German states with the highest tularemia incidence (34). All isolates identified in this study belonged to *F*. *tularensis* subsp. *holarctica*. Other subspecies have been reported sporadically in different regions of Germany and Europe (36).

Viral diseases were also relevant causes of death in lagomorphs. European brown hare syndrome (EBHS) was detected exclusively in brown hares and was characterized by acute hepatic necrosis, splenomegaly and multifocal hemorrhages, particularly affecting the liver and lungs. The peracute to acute disease course frequently resulted in sudden death. Diagnosis was based on characteristic pathological findings and was confirmed by quantitative PCR (qPCR) targeting the EBHS virus. EBHS represents an important differential diagnosis to tularemia, as both conditions may present without preceding clinical signs. Rabbit hemorrhagic disease (RHD) occurred only in European rabbits and was diagnosed based on characteristic pathological findings combined with virological confirmation. Both, EBHS and RHD, have substantial implications for population dynamics. Their repeated detection indicates persistence in southern Germany, justifying inclusion in future monitoring programs (37, 38).

Canine distemper virus (CDV) represented another major infectious cause of death, primarily affecting foxes, raccoons, and martens, with a regional concentration of cases in the Ravensburg area. This spatial aggregation is driven by ecological and methodological factors. High carnivore densities in urban and suburban areas facilitate virus transmission, while active local surveillance increases case detection compared to regions with less systematic monitoring (39, 40). Although the number of confirmed cases was relatively low, this is likely due to diagnostic practice. Suspected cases are often screened using indicator animals without full necropsy, limiting the detection of co-infections or concurrent pathological conditions. *CDV* is highly contagious, affects a broad host range, and poses spillover risks to domestic dogs. In Germany, no systematic wildlife vaccination campaigns exist. Vaccination of domestic dogs and ferrets is recommended as a core measure by the Standing Vaccination Commission for Veterinary Medicine (StIKo Vet) but remains voluntary (41). The absence of vaccination programs for CDV, in contrast to rabies control, leaves wild carnivore populations highly susceptible, emphasizing the importance of consistent vaccination in domestic animals and surveillance in wildlife.

Parasitic diseases also contributed substantially to mortality. Sarcoptic mange (*Sarcoptes scabiei*) in foxes caused generalized infestations characterized by alopecia, hyperkeratosis, secondary infections, and progressive emaciation. Severely affected animals may increasingly forage near human settlements, particularly during winter, where anthropogenic food sources are more accessible, thereby elevating the risk of pathogen transmission to humans (42). Sporadic crossover infections in dogs have been reported (43). Detection rates were particularly high in suburban areas, likely reflecting higher population densities and closer interspecific contact. Parasitic diseases were also documented in other species, such as roe deer, predominantly as gastrointestinal strongylid nematodes and pulmonary lungworm infestations resulting in chronic emaciation, anemia, and ultimately death.

Although echinococcosis was not a frequent direct cause of death, its detection in foxes is highly relevant from a One Health perspective. The red fox serves as the main definitive host of *Echinococcus multilocularis*, the causative agent of human alveolar echinococcosis. In Germany, human incidence is highest in the southern federal states. According to the Robert Koch Institute, 263 human cases were reported nationwide between 2023 and 2024, with more than 40% originating from Baden-Wuerttemberg and Bavaria (44). Southern Germany is an endemic area for alveolar echinococcosis due to a long-established sylvatic cycle of *E*. *multilocularis* between foxes and small rodents, supported by favorable landscape and climatic conditions that sustain a high environmental parasite biomass and continuous human exposure (45). Due to the long incubation period and late clinical manifestation, human cases are often diagnosed at advanced stages. Continuous wildlife surveillance therefore represents an essential early warning system for zoonotic risks (46).

### 4.5 Trauma as the second leading cause of death

Traumatic events represented the second most frequent cause of death identified in this study, with blunt force trauma predominating. Typical pathological findings included multiple fractures such as serial rib fractures, hemothorax, hemoabdomen, pulmonary hemorrhage, and rupture of internal organs. Traffic collisions were the most common traumatic scenario and affected several species, including wild boars, European wildcats, Eurasian beavers and pine- and stone martens. In 56% of blunt trauma cases, the exact event could not be fully reconstructed due to advanced autolysis or limited contextual information. However, the distribution and combination of lesions allowed reliable attribution to blunt force trauma.

Traffic related trauma represents a shared risk at the human-wildlife interface. Collision risk is influenced by traffic volume, road design and seasonal animal movement patterns (47). These factors act across species boundaries and reflect broader effects of anthropogenic transport systems. Mitigation measures such as wildlife overpasses, reduced speed zones in high-risk areas and awareness strategies have been shown to reduce wildlife vehicle collisions and improve overall traffic safety (48).

Among the affected species, the European wildcat is particularly vulnerable. Road traffic constitutes a major mortality factor and may disrupt dispersal corridors, thereby reducing gene flow between subpopulations. Regional studies reported mortality rates of up to 2.7 individuals per 100 km of road per year. Genetic analyses identified road density as the strongest barrier to gene flow, exceeding the effects of major highways (49). Evidence suggests that structurally intensive mitigation measures outperform low-cost deterrents, such as light- or sound-based systems. Fencing alone has been shown to reduce wildlife-vehicle collisions by approximately 40% in large mammals and by up to 83% in European wildcats (50, 51).

Sharp trauma was less frequent and mainly consisted of bite wounds. These injuries were attributable either to natural predators (e.g., foxes) or to domestic animals such as dogs and cats. Roe deer and bats were particularly affected. In urban and suburban environments, distinguishing natural predation from anthropogenic causes remains challenging, as free-ranging pets contribute substantially to injury risks (52, 53). Hunting-related injuries were also identified, including 30 gunshot cases. Some were consistent with legal hunting, while others suggested illegal persecution. A small number of fatalities were associated with mowing machinery. These cases primarily affected juvenile animals and are well documented in agriculture landscapes, particularly in roe deer fawns during mowing periods (54). Several preventive approaches have been evaluated. In a field study conducted in 2019 by the Bavarian State Research Center for Agriculture (LfL) in cooperation with the Hunting Authority (“Obere Jagdbehoerde”), different wildlife detection methods were tested prior to mowing. Portable handheld wildlife detectors (“Wildretter”) achieved a detection success of approximately 67%, whereas drone-based systems detected animals in only 31% (55). Targeted awareness campaigns and adapted mowing practices therefore remain essential as preventive tools (54). Overall, anthropogenic influences accounted for a substantial proportion of trauma-related deaths. This is consistent with studies from Central Europe, where traffic, agriculture, fencing, and wind energy are major mortality drivers (49, 56–61). Road traffic represents a multifaceted threat, causing direct mortality and contributing to habitat fragmentation, reduced genetic diversity, and altered population dynamics. The strong association between road infrastructure and wildlife mortality highlights the need for protective measures. Such strategies support biodiversity conservation and improve human traffic safety. An integrated approach combining ecological knowledge, traffic planning and public awareness is essential for sustainable coexistence between humans and wildlife.

### 4.6 Impact of the COVID-19 pandemic

Case numbers declined markedly during COVID-19 lockdowns (62). This decrease does not indicate a true reduction in wildlife mortality alone but reflects a combination of reduced surveillance activity and lower traffic volume. Movement restrictions, limited laboratory access and temporary reprioritization of resources led to fewer carcass submissions and reduced diagnostic capacity. At the same time, decreased human mobility was associated with reduced traffic density and coincided with a decline in wildlife-vehicle collisions. Studies from the United Kingdom reported reductions of up to 80% wildlife-vehicle collisions during lockdown periods, while citizen science data from Austria and Poland documented fewer recorded roadkill events linked to decreased travel and vehicle numbers (63–65). Similar disruptions of wildlife health monitoring and biodiversity recording systems were reported across Europe and globally during the COVID-19 pandemic, including reduced surveillance activities and substantial declines in biodiversity data collection (66, 67). Local reductions of more than 50% in recorded hedgehog roadkill further illustrate that the observed declines reflects both reduced traffic exposure and changes in reporting intensity (65). These findings highlight the importance of considering surveillance effort, sampling intensity, and traffic dynamics for the interpretation of temporal trends in wildlife mortality data.

### 4.7 Significance of the ICD-11 scheme

The application of ICD-11 (International Classification of Diseases, 11th Revision) enabled a standardized and internationally comparable classification of wildlife mortality in this study. Its modular structure supports consistent coding and allows representation of complex disease constellations including co-infections. This facilitates comparison with human health data and supports One Health oriented analyzes. Similar conclusions were reported by Marchetti et al. (2022), who identified ICD-11 as a useful tool for harmonizing animal and human health records and support cross-sector surveillance (8). However, ICD-11 was originally developed for human medicine and its transfer to veterinary pathology poses several challenges. Certain disease entities and anatomical differences are not explicitly represented. Wildlife cases often lack detailed clinical histories or exposure data, which limits precise assignment within a system designed for clinically well documented patients. The distinction between primary causes of death and contributing conditions is also less clear in postmortem wildlife investigations, particularly in cases involving trauma, advanced autolysis, or multiple interacting factors. These limitations require careful interpretation and transparent documentation when ICD-11 is applied outside its original clinical context.

Despite these constraints, ICD-11 can function as a methodological interface between veterinary and human medicine when applied in a structured and critical manner. Wildlife mortality data generated using such standardized approaches can contribute to international reporting systems and strengthen early detection of zoonotic risks.

Beyond its practical application, ICD-11 also served in this study as an illustrative framework highlighting the need for a standardized and interoperable classification system in veterinary medicine. Unlike human medicine, veterinary pathology currently lacks a universally accepted system for coding causes of death across species and regions. This limits comparability between studies, hampers large scale surveillance and constrains integration into One Health monitoring structures (68). The use of ICD-11 in this study demonstrates both the feasibility of standardized coding and the necessity for a veterinary adapted classification system that accommodates species specific pathology while remaining compatible with human health frameworks.

## 5 Conclusion

The systematic investigation of causes of death in wild mammals provides insight into health risks, environmental conditions, and anthropogenic pressures affecting wildlife populations. Such data are particularly relevant for the One Health framework, as wild mammals act as sentinels of zoonotic risks, environmental changes, and emerging pathogens. However, the interpretability of wildlife mortality data strongly depends on how surveillance systems are structured. More representative and less selective monitoring would improve population-level risk assessment, at the same time, comprehensive postmortem examination of all deceased wildlife is logistically and economically unrealistic and has not been implemented even in well-funded wildlife health systems.

The use of the ICD-11 enabled a standardized and internationally comparable classification of wildlife mortality and allowed alignment with human health data structures. The result demonstrates the strong impact of road traffic and other anthropogenic factors on wildlife mortality. This underlines the need for targeted mitigation measures, including wildlife crossings, surveillance programs and vaccination of domestic reservoir hosts. Necropsy based monitoring can therefore support One Health objectives, if it is applied in a systematic and epidemiologically interpretable manner. Strengthened and harmonized surveillance would benefit both biodiversity conservation and integrated risk assessment at the human- animal- environment interface.

## Data Availability

The original dataset consists of necropsy records (hard copies of necropsy forms, partially hand-written and not completely digitalized) of different institutions. These original data are restricted due to legal and ethical constraints and data ownership by the competent authorities. Individual case-level data and original necropsy reports can, therefore, not be released publicly. However, we can provide the de-identified data on individual investigated cases upon request. Requests to access these datasets should be directed to Melanie Nowak (née Henker), melanie.henker@patho.vetmed.uni-muenchen.de.
