# Supplementary material for: Retrospective multicenter analysis of causes of death in wild mammals in Southern Germany (2019–2023)
Source: Front Vet Sci. 2026 Apr 21;13:1805419. doi: 10.3389/fvets.2026.1805419 (PMC13139095; doi:10.3389/fvets.2026.1805419)
Supplement: Supplementary file 1 [file Table_1.DOCX]

Table S1 Frequency and percentage of the causes of death by sex, age, and season (irrespective of species).

| **Nontraumatic Death** | | | | | |
| --- | --- | --- | --- | --- | --- |
| **Category** | **Infectious/ Inflammatory Disease** | **Metabolic/ Toxic Diseases** | **Other (Cardiovascular-, Congenital Diseases, Neoplasm)** | **Nontraumatic Death of Unknown Origin** | **Total** |
| **Sex** |  | | | | |
| Male | 652 (49%) | 33 (43%) | 17 (40%) | 9 (26%) | 711 (48%) |
| Female | 593 (45%) | 35 (45%) | 23 (53%) | 15 (43%) | 666 (45%) |
| Unknown | 77 (6%) | 9 (12%) | 3 (7%) | 11 (31%) | 100 (7%) |
| Other (DSD)* | 1 (0.1%) | 0 | 0 | 0 | 1 (0.1%) |
| **Age** |  | | | | |
| Adult + Geriatric | 790 (60%) | 41 (53%) | 26 (60%) | 16 (46%) | 873 (59%) |
| Juvenil | 156 (12%) | 5 (6%) | 3 (7%) | 7 (20%) | 171 (12%) |
| Neonates | 173 (13%) | 21 (27%) | 6 (14%) | 4 (11%) | 204 (14%) |
| Unknown | 204 (15%) | 10 (13%) | 8 (19%) | 8 (23%) | 230 (16%) |
| **Season** |  |  |  |  |  |
| Spring | 385 (29%) | 37 (48%) | 15 (35%) | 6 (17%) | 443 (30%) |
| Summer | 229 (17%) | 17 (22%) | 10 (23%) | 12 (34%) | 268 (18%) |
| Autumn | 422 (32%) | 8 (10%) | 7 (16%) | 9 (26%) | 446 (30%) |
| Winter | 287 (22%) | 15 (19%) | 11 (25%) | 8 (23%) | 321 (22%) |
| **Traumatic Death** | | | | | |
| **Category** | **Blunt force trauma** | **Sharp forced trauma** | **Other trauma** |  | **Total** |
| **Sex** |  | | | | |
| Male | 236 (48%) | 44 (39%) | 18 (51%) |  | 298 (47%) |
| Female | 198 (40%) | 51 (45%) | 15 (43%) |  | 264 (41%) |
| Unknown | 56 (11%) | 19 (16%) | 2 (6%) |  | 77 (12%) |
| **Age** |  | | | | |
| Adult + Geriatric | 314 (64%) | 70 (61%) | 21 (60%) |  | 405 (63%) |
| Juvenil | 54 (11%) | 9 (8%) | 8 (23%) |  | 71 (11%) |
| Neonates | 52 (11%) | 13 (11%) | 3 (9%) |  | 68 (11%) |
| Unknown | 70 (14%) | 22 (19%) | 3 (9%) |  | 95 (15%) |
| **Season** |  | | | | |
| Spring | 126 (26%) | 39 (34%) | 10 (29%) |  | 175 (27%) |
| Summer | 132 (27%) | 38 (33%) | 8 (23%) |  | 178 (28%) |
| Autumn | 131 (27%) | 19 (17%) | 5 (14%) |  | 155 (24%) |
| Winter | 101 (21%) | 18 (16%) | 12 (34%) |  | 131 (21%) |

*Disorders of Sexual Development
