# Supplementary material for: Retrospective multicenter analysis of causes of death in wild mammals in Southern Germany (2019–2023)
Source: Front Vet Sci. 2026 Apr 21;13:1805419. doi: 10.3389/fvets.2026.1805419 (PMC13139095; doi:10.3389/fvets.2026.1805419)
Supplement: Supplementary file 2 [file Table_2.DOCX]

Table S2 Frequency and percentage of anthropogenic related trauma (irrespective of species).

| **Category** | **Anthropogenic Trauma** |
| --- | --- |
| **Sex** | |
| Male | 118 (44%) |
| Female | 119 (44%) |
| Unknown | 32 (12%) |
| **Age** | |
| Adult + Geriatric | 174 (65%) |
| Juvenil | 23 (9%) |
| Neonates | 33 (12%) |
| Unknown | 39 (14%) |
| **Season** | |
| Spring | 74 (28%) |
| Summer | 68 (25%) |
| Autumn | 73 (27%) |
| Winter | 54 (20%) |
